# Multicolour imaging with spectral photon-counting CT: a phantom study

**DOI:** 10.1186/s41747-018-0063-4

**Published:** 2018-10-17

**Authors:** Salim Si-Mohamed, Daniel Bar-Ness, Monica Sigovan, Valérie Tatard-Leitman, David P. Cormode, Pratap C. Naha, Philippe Coulon, Lucie Rascle, Ewald Roessl, Michal Rokni, Ami Altman, Yoad Yagil, Loic Boussel, Philippe Douek

**Affiliations:** 1 University Claude Bernard Lyon1, CREATIS, CNRS UMR 5220, INSERM U1206, INSA-Lyon, Lyon, France; 2 Radiology Department, Hospices Civils de Lyon, Lyon, France; 3 Global Advanced Technologies, CT, Philips, Haifa, Israel; 4 Department of Radiology, University of Pennsylvania, Philadelphia, PA USA; 5 CT Clinical Science, Philips, Suresnes, France; 6 Philips GmbH Innovative Technologies, Research Laboratories, Hamburg, Germany

**Keywords:** Gadolinium, Gold, Iodine, Phantoms (imaging), Tomography (x-ray computed)

## Abstract

**Background:**

To evaluate the feasibility of multicolour quantitative imaging with spectral photon-counting computed tomography (SPCCT) of different mixed contrast agents.

**Methods:**

Phantoms containing eleven tubes with mixtures of varying proportions of two contrast agents (i.e. two selected from gadolinium, iodine or gold nanoparticles) were prepared so that the attenuation of each tube was about 280 HU. Scans were acquired at 120 kVp and 100 mAs using a five-bin preclinical SPCCT prototype, generating conventional, water, iodine, gadolinium and gold images. The correlation between prepared and measured concentrations was assessed using linear regression. The cross-contamination was measured for each material as the root mean square error (RMSE) of its concentration in the other material images, where no signal was expected. The contrast-to-noise ratio (CNR) relative to a phosphate buffered saline tube was calculated for each contrast agent.

**Results:**

The solutions had similar attenuations (279 ± 10 HU, mean ± standard deviation) and could not be differentiated on conventional images. However, a distinction was observed in the material images within the same samples, and the measured and prepared concentrations were strongly correlated (R^2^ ≥ 0.97, 0.81 ≤ slope ≤ 0.95, -0.68 ≤ offset ≤ 0.89 mg/mL). Cross-contamination in the iodine images for the mixture of gold and gadolinium contrast agents (RMSE = 0.34 mg/mL) was observed. CNR for 1 mg/mL of contrast agent was better for the mixture of iodine and gadolinium (CNR_iodine_ = 3.20, CNR_gadolinium_ = 2.80) than gold and gadolinium (CNR_gadolinium_ = 1.67, CNR_gold_ = 1.37).

**Conclusions:**

SPCCT enables multicolour quantitative imaging. As a result, it should be possible to perform imaging of multiple uptake phases of a given tissue/organ within a single scan by injecting different contrast agents sequentially.

## Key points

- SPCCT is capable of discriminating between two contrast agents within the same image location via K-edge imaging.
- SPCCT K- enables the discrimination between either one K-edge material and iodine such as gadolinium, or two K-edge materials, such as gold and gadolinium.
- SPCCT can qualitatively and quantitatively separate either gadolinium and iodine or gadolinium and gold with good accuracy (offsets between -0.68 and 0.89 mg/mL, R^2^ ≥ 0.97).

## Background

Since the emergence of clinical dual-energy computed tomography (DECT) and spectral photon-counting computed tomography (SPCCT), there has been an increasing interest in using these systems to discriminate between different contrast agents, based on their specific x-ray attenuation characteristics [1–9]. For example, DECT has recently been used for *in vivo* depiction of the small bowel wall and for the differentiation between a vascular and an enteric injury, using a combination of an intravenous iodinated contrast agent with an oral tantalum- or tungsten-based contrast agent [10] or oral bismuth [11]. However, because DECT is limited by its sampling capacity of only two predefined energy spectra, typically high (140 kVp) and low (80 kVp) [5, 12, 13], the attenuation model used for reconstruction is restrained to the material decomposition of only two base images [5, 14]. Any other material is represented as an equivalent ratio of the two base materials. Moreover, the DECT material decomposition scheme does not contain the K-edge discontinuity, which has to be determined using data from an additional energy [5, 15, 16].

Unlike DECT, SPCCT uses energy-resolving detectors that can simultaneously sample the energy spectrum at multiple regions. This allows a higher spectral resolution, enabling the identification of material-specific spectral characteristics, such as the K-edge signature of the contrast agent [5, 15–18]. This is particularly interesting as the K-edge energies of contrast agents that contain heavy elements such as gadolinium, ytterbium, bismuth or gold are within the clinical x-ray tube spectrum [1, 17, 19–21]. The material decomposition can then be extended to include the K-edge of the contrast agents as a separate basis, allowing for multicolour imaging [8, 22, 23]. However, despite its high spectral resolution, it is still unknown whether the SPCCT system can distinguish and quantify two contrast agents when they are mixed within the same volume (either one with high and the other with low K-edge energy or both with high K-edge energies).

In this study, we proposed to fill this gap in knowledge by performing multicolour imaging with SPCCT and detect as well as quantify iodine, gadolinium and gold in different mixtures, with a single scan.

## Methods

### Phantom preparation

**Fig. 1:**
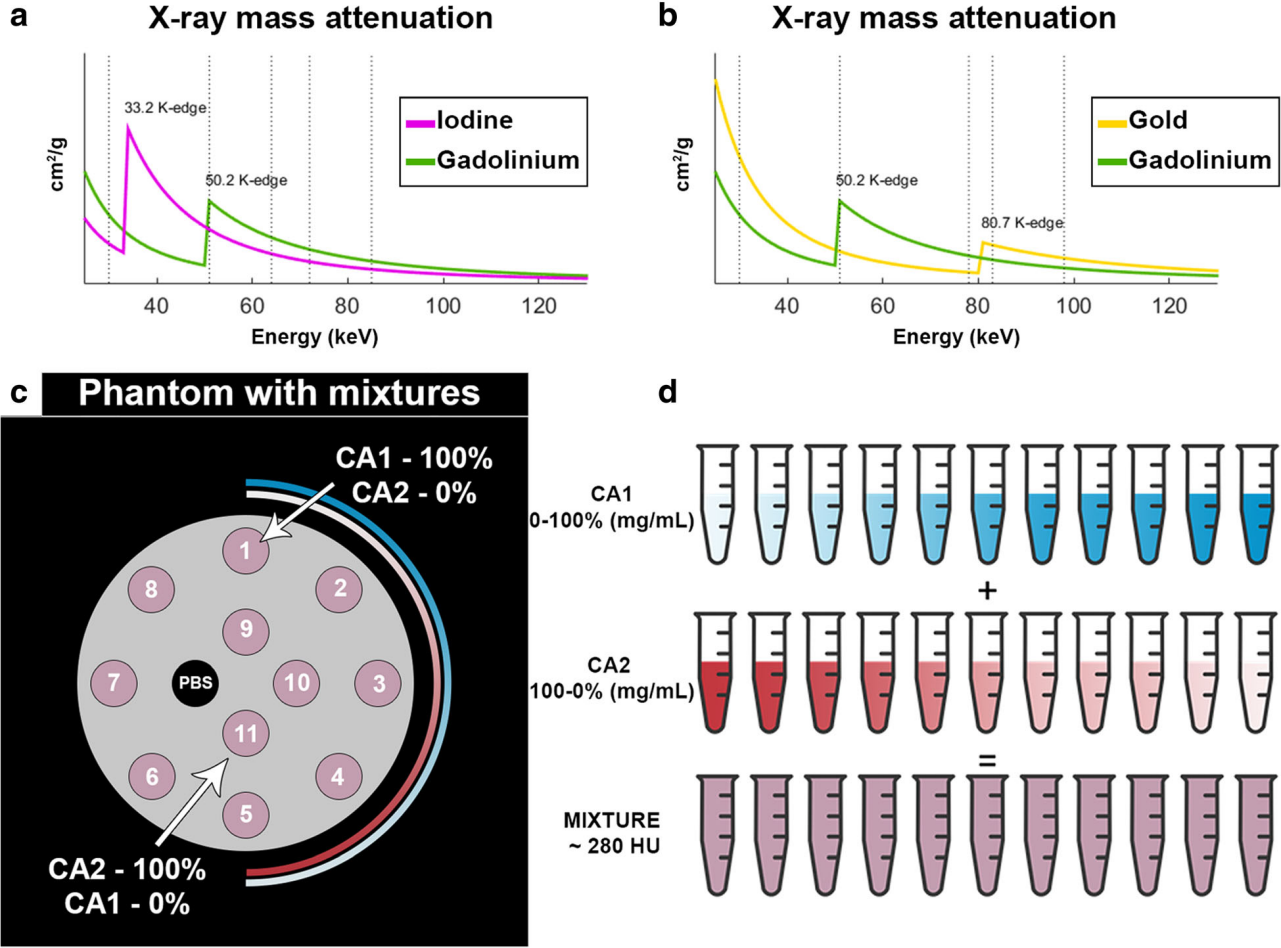
Representation of the K-edge energies and thresholds (*dotted lines*) used for the characterisation of each element. x-ray mass attenuations of iodine (*purple*) and gadolinium (*green*) (**a**), as well as gold (*yellow*) and gadolinium (*green*) (**b**) are shown with *solid lines*. **c**, **d** Eleven tubes of mixed contrast agents were prepared. The solutions were organised in a spiral, such that contrast agent 1 (in *blue*) was prepared at a decreasing concentration while contrast agent 2 (in *red*) was prepared at an increasing concentration. The tubes in the phantom have the same colour on the schema because of their same-targeted CT attenuation values (~ 280 HU)

A custom-made polyoxymethylene cylindrical phantom with a diameter of 13 cm and 12 holes with a 1.5-cm diameter was used. The contrast agents used in this study were: gold nanoparticles (AuNP; 65 mg/mL, size 18 nm, synthesised in-house) [24]; an iodinated contrast agent (Iomeron 400 mg/mL, Bracco, Milan, Italy); and a gadolinium chelate (MultiHance 0.5 mmoL/mL, 78.625 mg/mL, Bracco, Milan, Italy). The samples were loaded into the phantom using 1.5-mL polypropylene centrifuge tubes. Two sets of 11 tubes were prepared, each containing two contrast agents diluted in phosphate buffer saline (PBS) and mixed in varying proportions, as previously described [8, 21] (Fig. 1c, d). The mixed agents were either gadolinium mixed with iodine or gadolinium mixed with gold. Eleven samples each of unmixed AuNP, gadolinium or iodine were also prepared in order to examine the effects of mixing the contrast agents on the measurement of their concentrations. The proportions of each pair of contrast agents were adjusted using a proper MATLAB code that allows calculations based on data obtained from imaging unmixed contrast agents. The concentrations (mg/mL) of the mixtures depended on the corresponding attenuation at 120 kVp. The target attenuation for each solution was chosen within the clinical standard for CT angiography applications, i.e. 280 Hounsfield units (HU) approximately [25]. Hence, the concentrations of gadolinium, AuNP and iodine were in the range of 0–7.45 mg/mL, 0–10.4 mg/mL and 0–8 mg/mL, respectively. The contrast agent concentrations are presented in Table 1.

**Table 1:** Concentrations of the three different contrast agents used in mixtures or individually diluted to obtain the six sets of 11 tubes

Tube ID	Mixed gold and gadolinium		Mixed iodine and gadolinium		Gold only	Gadolinium only	Iodine only
	Gold (mg/mL)	Gadolinium (mg/mL)	Iodine (mg/mL)	Gadolinium (mg/mL)	Gold (mg/mL)	Gadolinium (mg/mL)	Iodine (mg/mL)
1	0	7.46	8	0	0	0	8
2	0.98	6.67	7.2	0.79	0.98	0.79	7.2
3	1.95	5.89	6.4	1.57	1.95	1.57	6.4
4	3.25	5.1	5.6	2.36	3.25	2.36	5.6
5	4.22	4.32	4.8	2.75	4.22	2.75	4.8
6	5.2	3.53	4	3.53	5.2	3.53	4
7	6.17	2.75	3.2	4.32	6.17	4.32	3.2
8	7.15	2.36	2.4	5.1	7.15	5.1	2.4
9	8.13	1.57	1.6	5.89	8.13	5.89	1.6
10	9.43	0.79	0.8	6.67	9.43	6.67	0.8
11	10.4	0	0	7.07	10.4	7.07	0

### Spectral photon-counting computed tomography

The modified clinical base, small field of view (FOV) SPCCT prototype system (Philips Healthcare, Haifa, Israel) is equipped with a conventional x-ray tube that can be set with a tube voltage at 80, 100 or 120 kVp and tube current values in the range of 10–100 mA. The system is based on a semi-conductor detector technology operated in single photon-counting mode with energy discrimination [15, 26]. The system is operated as a nine-row scanner with an effective z-collimation of about 2.5 mm in the isocentre and an imaging FOV of 168 mm in-plane. Axial scans > 360° were performed at a tube current of 100 mA and a tube voltage of 120 kVp (weighted CT dose index = 0.74 mGy) with a scanner rotation time of 1 s and 2400 projections per rotation. Note that the CT dose index on SPCCT prototype is higher than what we expect on a future clinical scanner for the same kVp and mAs because the current creates very small z-collimation resulting in low dose efficiency due to the small useful part of the beam relative to the penumbra part. Each pixel in each projection reading consisted of a group of five integer numbers, representing the number of crossings (from low to high) of the analogue pulse signal of predefined energy thresholds. For low flux and negligible pile-up, these numbers corresponded to the counts in single-ended pulse-height windows that resulted from the convolution of the x-ray spectrum and the detector response function. Due to the small FOV and the small z-collimation of the prototype, scatter effects were much smaller than for clinical CT. Thus, the detector of this prototype was not equipped with an anti-scatter grid. This system had five thresholds that could be adjusted in order to allow photon energy-based discrimination of an element. In the cases of gadolinium and gold, we set two thresholds just below and above their K-edges, at 50.2 keV and 80.7 keV, respectively, as was detailed in the study by Roessl et al. [27]. One additional energy threshold served as a noise threshold and was set to 30 keV. Hence, the energy thresholds were set at 30, 51, 64, 72 and 85 keV for the gadolinium study, at 30, 53, 78, 83 and 98 keV for the gold study, at 30, 51, 78, 83 and 98 keV for the gold and gadolinium mixture study, and at 30, 51, 64, 72 and 85 keV for the iodine and gadolinium mixture study (Fig. 1a, b). The energy thresholds for the iodine study were set at 30, 51, 64, 72 and 85 keV, even though iodine could not be efficiently detected by the K-edge technique due to the low numbers of photons around its K-edge energy (33.2 keV) [28].

### Data acquisition and spectral image reconstruction

All solutions were scanned in the same phantom using the SPCCT prototype. The same SPCCT scan protocol was used, i.e. axial scan at a tube voltage of 120 kVp, tube current of 100 mAs and FOV of 160 mm. For each pixel, a maximum likelihood estimator was used to derive an equivalent water-thickness per pixel from the photon counts in the five energy bins. Conventional images were then reconstructed from the water-thickness equivalent sinograms using a filtered back-projection algorithm. A material decomposition based on a forward projection model (per detector) and maximum likelihood, and literature data provided by the National Institute of Standards and Technology (Gaithersburg, MD, USA) [29], was used for the energy-dependent attenuation of the base materials. For each phantom configuration, a specific material basis consisting of at least water and iodine and—in case of their presence—gadolinium and/or gold was chosen. For example, a three-material water–iodine–gadolinium basis was used for the gadolinium–iodine study while a four-material water–iodine–gold–gadolinium basis was selected for the gadolinium–gold mixture study. The five multi-energy sinograms were then decomposed with a maximum-likelihood algorithm [16] into a set of up to four base material sinograms for the given material basis. Finally, the base material sinograms were individually reconstructed. The sinograms were individually reconstructed with a wedge reconstruction algorithm [30, 31] on a 0.4 × 0.4 × 0.25 mm^3^ voxel grid. The reconstructed volume was then averaged over all eight slices to reduce noise so that the images analysed were 2-mm thick. Hence, for each of the contrast agent mixtures, a distinct set of contrast material images appropriate for each contrast agent combination was generated within a few minutes for each slice, i.e. water, iodine, gadolinium and gold images. The water and iodine images were reconstructed for each solution no matter the content; the gadolinium and gold images were reconstructed only in the presence of the material. The images were then analysed with further post-processing using deringing and smoothing of the contrast material images with a Gaussian kernel with a 2-pixel radius and sigma.

### Image analysis

Image analysis was performed using MATLAB (R2015a, MathWorks, Inc.). The samples were automatically detected on conventional images and circular regions of interest (ROIs) were automatically drawn in the middle of each tube. The same ROIs were used on all images generated on a given dataset and mean and standard deviation were computed for each ROI. Linear regression was performed between the prepared and measured concentrations for all solutions. The cross-contamination of the material decomposition, i.e. the amount of a measured material in other material image (e.g. amount of gadolinium and gold measured in water and iodine images), was measured for each material as the root mean square error (RMSE) of its concentration in the other basis materials’ ROIs, where no signal would be expected in the iodine image and where a mean value of 1000 mg/mL would be expected in the water image.

For each contrast agent in both unmixed and mixed solutions, the CNR relative to a homogeneous PBS tube was calculated according to the following equation:

$$ \mathrm{CNR}=\frac{\mid {\mathrm{mean}\ \mathrm{concentration}}_{\mathrm{tube}\ \mathrm{with}\ \mathrm{contrast}\ \mathrm{agent}}-{\mathrm{mean}\ \mathrm{concentration}}_{\mathrm{tube}\ \mathrm{with}\ \mathrm{PBS}\ \mathrm{only}}\mid }{{\mathrm{Standard}\ \mathrm{deviation}}_{\mathrm{tube}\ \mathrm{with}\ \mathrm{PBS}\ \mathrm{only}}} $$

### Statistical analysis

In order to assess the impact of mixing the contrast agents, a reliability analysis between the non-mixed and the mixed measured concentrations was performed using intraclass correlation coefficients (ICC) and their 95% confident intervals (CI). The statistical analyses were performed using SPSS statistical package version 24 (SPSS Inc., Chicago, IL, USA) based on a mean rating for each contrast agent (k = 3 e.g. Gd, Gd + AuNP, Gd + I), two-way mixed-effects model.

## Results

**Fig. 2:**
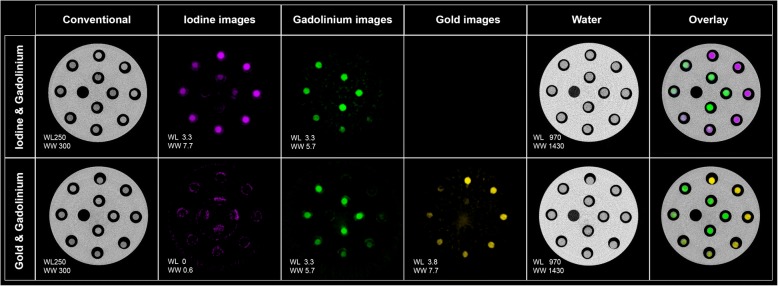
Spectral photon-counting images of conventional, iodine (*purple*), gadolinium (*green*), gold (*yellow*) and water for both sets of mixed contrast agents. *Top*: iodine–gadolinium mixture; *bottom*: gold–gadolinium mixture; conventional image units are HU and material images (water, iodine, gold, gadolinium) units are mg/mL. Note that no gold image was generated from the material decomposition process for the iodine–gadolinium mixture, whereas an iodine image was generated from the material decomposition process for the gadolinium–gold mixture

As expected from the experimental design, different solutions of mixed contrast agents could not be differentiated on conventional images as all tubes had similar attenuations (279 ± 10 HU). However, we found that the contrast agents were accurately identified in the material images and that the water images showed only the material-based water map in the solutions and in the phantom (Fig. 2).

**Fig. 3:**
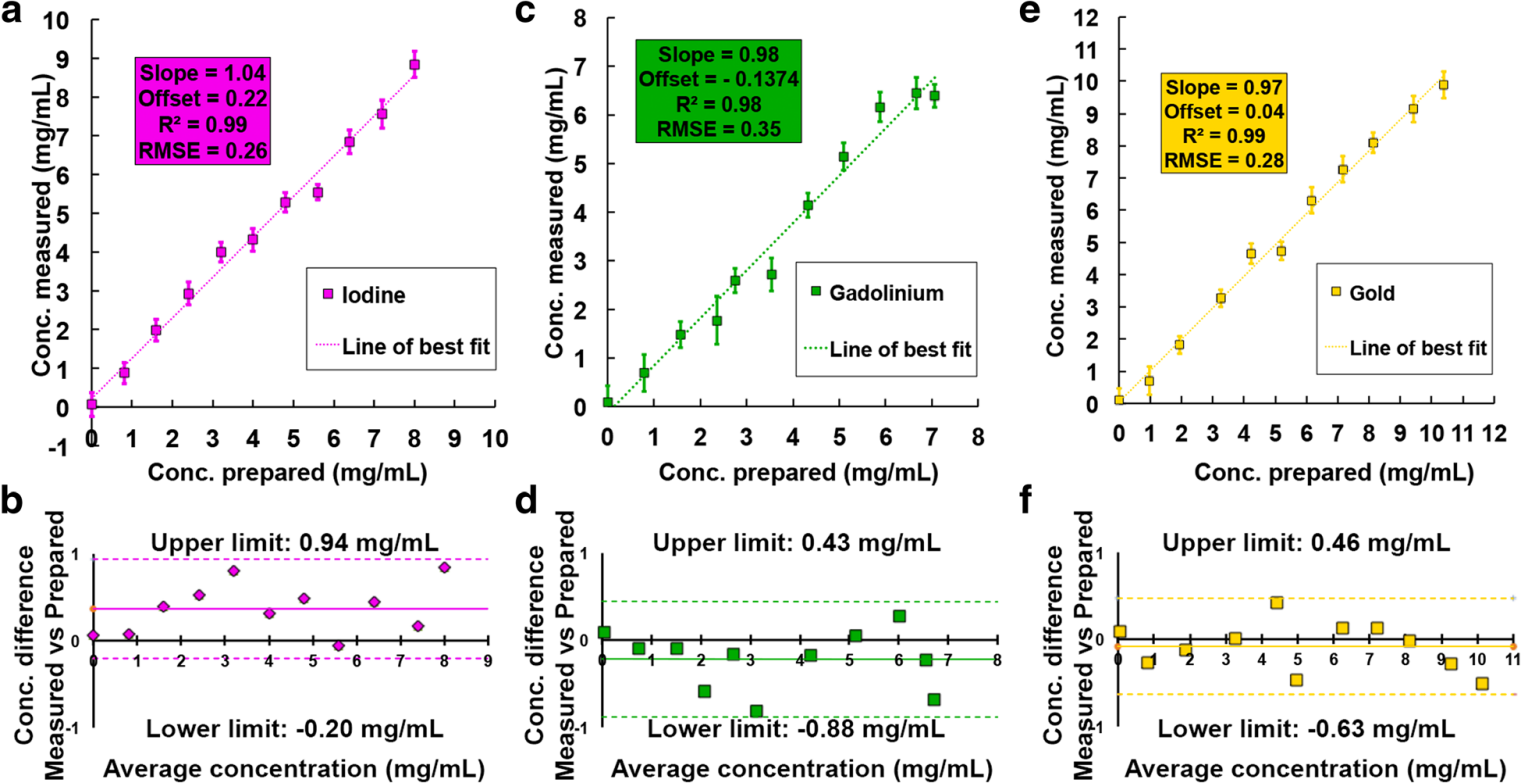
Measurements of the concentrations of contrast agents in the unmixed solutions. The concentrations of iodine (*purple*) (**a**, **b**), gadolinium (*green*) (**c**, **d**) and gold (*yellow*) (**e**, **f**) were measured in their respective unmixed solutions. **a**, **c**, **e** Linear regression; **b**, **d**, **f** Bland–Altman analysis. Note the linear correlations and the slope values approaching 1 for all contrast agents

**Fig. 4:**
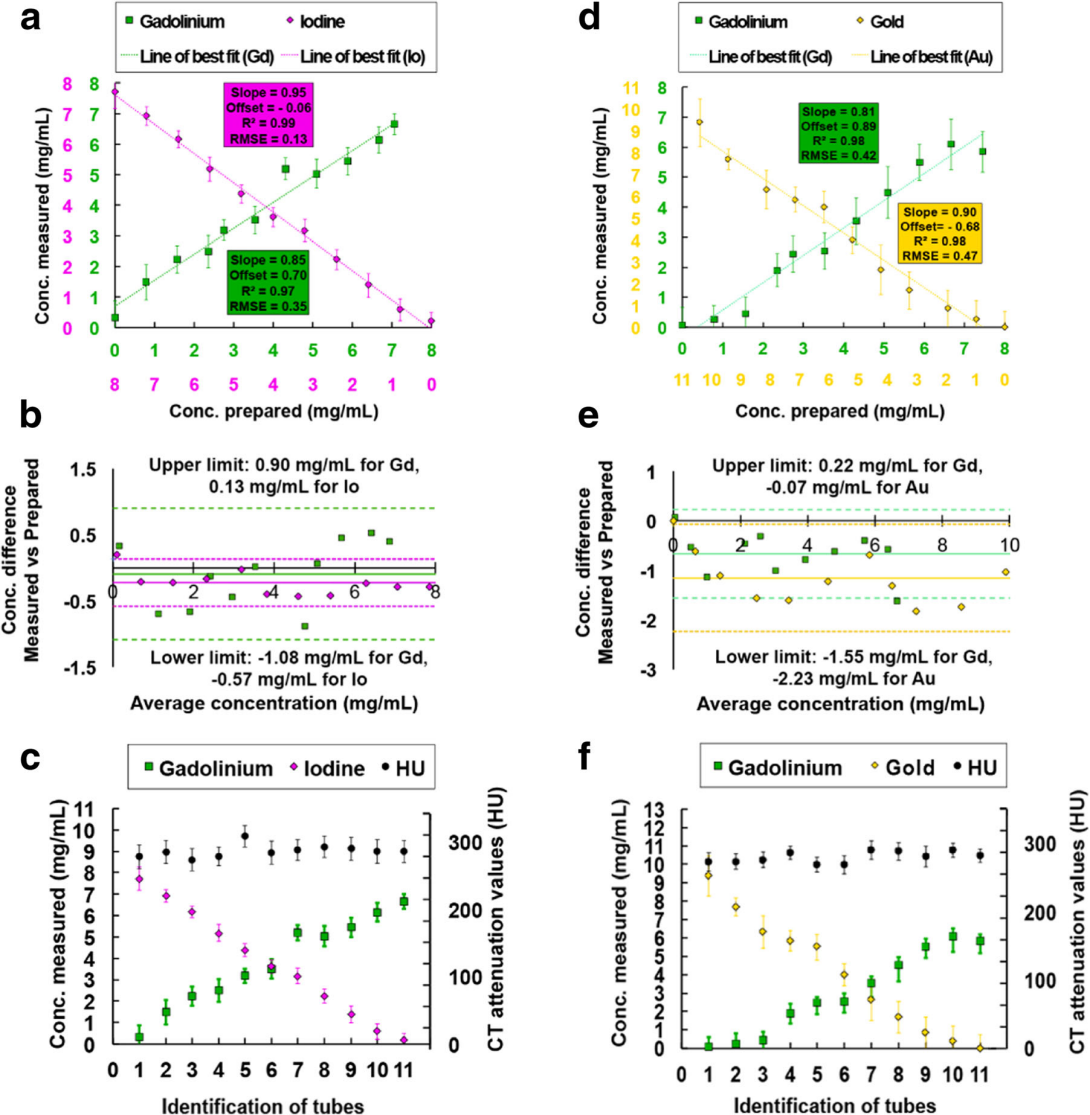
Measurements of the concentrations of contrast agents and attenuation values in the mixed solutions. The concentrations of each contrast agent were measured within the gadolinium–iodine (**a**, **b**, **c**) and gadolinium–gold (**d**, **e**, **f**) mixtures. **a**, **d** Linear regression; **b**, **e**: Bland–Altman analysis; **c**, **f**: graphs of the mean ± standard deviation of attenuation values and concentrations. Note that, as expected, the measured concentrations in the tubes varied inversely between the two mixed contrast agents

The correlations between the measured and prepared concentrations were strongly linear for all dilutions (all R^2^ ≥ 0.97). The slopes were close to 1 for the unmixed contrast agents solutions (0.98 ≤ slope ≤ 1.04), but were underestimated for the mixed contrast agents solutions (0.81 ≤ slope ≤ 0.95) (Figs. 3 and 4). The underestimations were slightly more marked for the gadolinium–gold mixture (gadolinium slope 0.81; gold slope 0.90), than for the iodine–gadolinium mixture (gadolinium slope 0.85; iodine slope 0.95) (Fig. 6). Additionally, the offset values were very low for single contrast agent solutions (-0.13 ≤ offset ≤ 0.22 mg/mL), but most of the offset values were significantly different from zero when the contrast agents were mixed (-0.68 ≤ offset ≤ 0.89 mg/mL) (Table 2 and Fig. 4).

**Table 2:** Linear regression coefficients of each reconstructed image from the different contrast agent mixtures and of the only individual contrast agent solutions

Image	Mixed gold and gadolinium		Mixed iodine and gadolinium		Gold only	Gadolinium only	Iodine only
	Gold	Gadolinium	Iodine	Gadolinium	Gold	Gadolinium	Iodine
Slope	0.90	0.81	0.95	0.81	0.97	0.98	1.04
Offset	-0.68	0.89	-0.06	0.88	0.04	-0.13	0.22
R^2^	0.98	0.98	0.99	0.98	0.99	0.98	0.99
RMSE	0.47	0.42	0.13	0.35	0.28	0.35	0.26
95% CI	0.83–1	0.86–1.1	0.96–1	0.77–1	0.9–0.97	0.82–1	0.99–1

*RMSE* root mean square error, *CI* confidence interval

The ICC analysis showed a significant linear relation between prepared and measured concentrations indicating an excellent degree of reproducibility between each of the contrast agent measurements (Table 2). Importantly, the linear relation indicates that offsets observed when contrast agents were mixed are not concentration-dependent. These quantitative results are summarised in Tables 2 and 3.

**Table 3:** Root mean square error (RMSE) coefficients of water and iodine reconstructed images from the different unmixed and mixed solutions

Image	Mixed gold and gadolinium		Mixed iodine and gadolinium	Gadolinium		Gold		Iodine
	Water	Iodine	Water	Water	Iodine	Water	Iodine	Water
RMSE	0.08	0.34	0.01	0.01	0.33	0.01	0.18	0.01

**Fig. 5:**
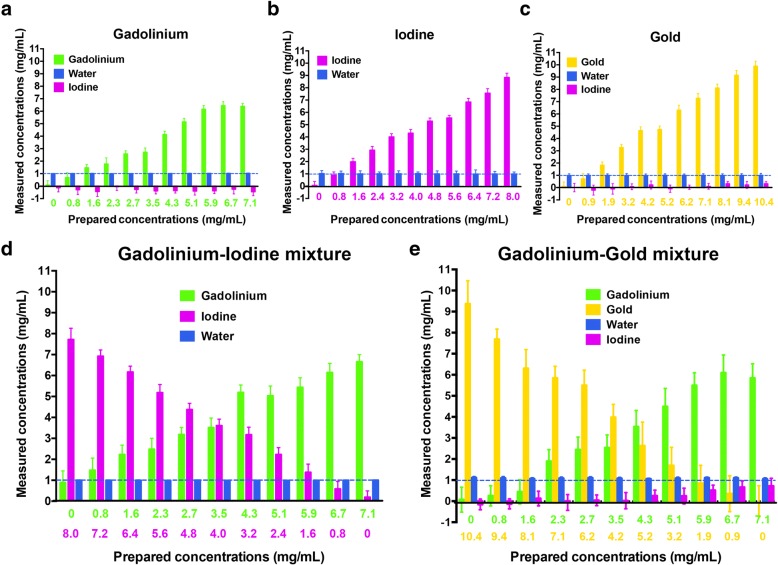
Cross-contamination in the contrast agent images. The *graph bars* show the concentrations (mean ± standard deviation) of water (*blue*), iodine (*purple*), gadolinium (*green*) and gold (*yellow*) measured by the system, in each contrast material images, as a function of the prepared concentrations within the unmixed (**a**, gadolinium; **b**, iodine; **c**, gold) and mixed (**d**, gadolinium–iodine; **e**, gadolinium–gold) solutions. The *blue dotted line* represents the concentration of water expected in each solution (1000 mg/mL). Note the slight cross-contamination in the iodine image for the gadolinium–gold mixture that increases with increasing concentration of gadolinium, but not gold

The water images did not show any cross-contamination for either the unmixed or the mixed solutions, as confirmed by the very low RMSE values (RMSE ≤ 0.08 mg/mL). Similarly, the iodine images did not show any cross-contamination for the unmixed solutions, despite a RMSE at 0.33 mg/mL for the gadolinium dilutions due to negative iodine measured concentrations. Conversely, the iodine images showed some cross-contamination for the gold–gadolinium mixture, as confirmed by a RMSE at 0.34 mg/mL and by the measured concentrations of iodine. The cross-contamination in iodine images increased with increasing concentrations of gadolinium but not gold (Table 3 and Fig. 5).

**Fig. 6:**
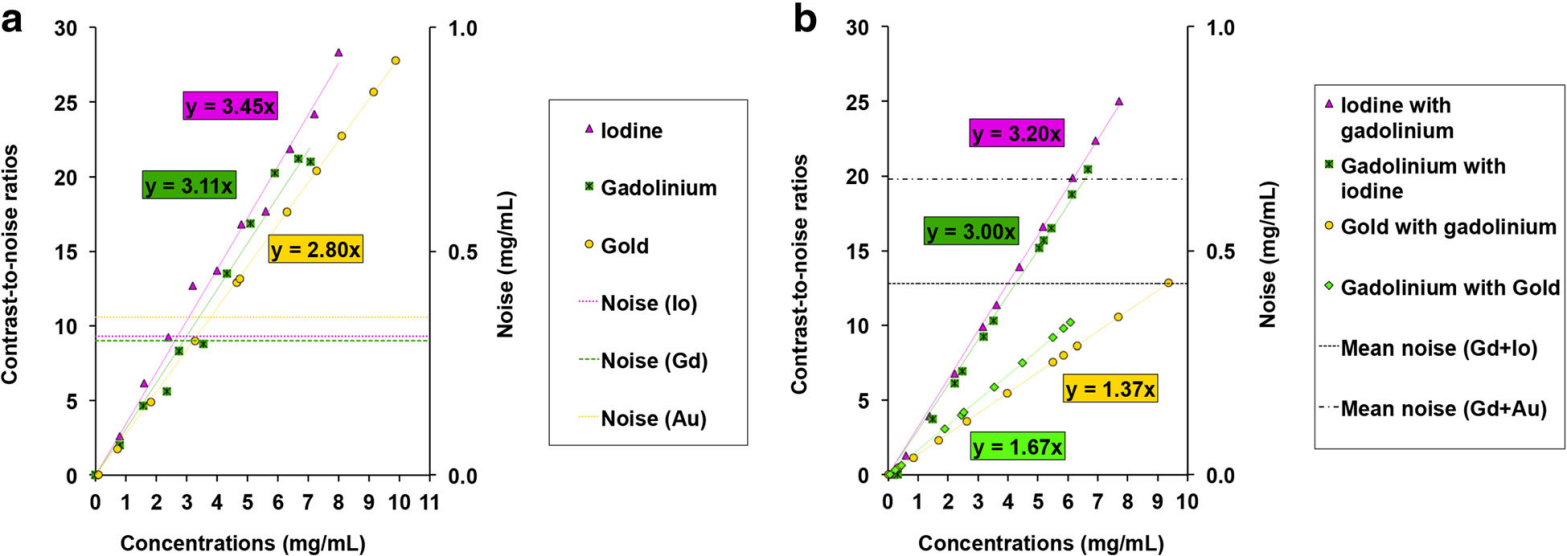
CNRs of the contrast agents. The *graphs* represent the CNR values depending on the concentration of the contrast agent and the noise values of the contrast agents measured in their respective contrast material maps within the unmixed (**a**) and mixed (**b**) solutions. The *dotted lines* represent the noise measured in a tube filled with PBS only within the contrast material images. Mean noise is the mean of the noise measured within the two contrast material images of each mixture

The CNR values observed for each contrast agent were higher for the unmixed solutions, in particular for the gold–gadolinium mixture (unmixed gold CNR slope 2.80; unmixed gadolinium CNR slope 3.11; gold–gadolinium mixture: gold CNR slope 1.37, gadolinium CNR slope 1.67) (Fig. 6). Furthermore, the CNR slope for unmixed iodine was better than for the gadolinium–iodine mixture (unmixed iodine CNR slope 3.45; gadolinium–iodine mixture: iodine CNR slope 3.20).

## Discussion

CT is an imaging modality with an excellent spatial resolution and fast acquisition times that has laid the foundation for SPCCT. In the present study, we demonstrate that SPCCT has the ability to separate two mixed contrast agents qualitatively and quantitatively. Indeed, the SPCCT energy-resolving detectors enable such spectral resolution that it is possible to distinguish K-edge signatures of different contrast agents, within the same voxel and at the same time. Consequently, separate quantity maps for each individual contrast agent can be generated. In addition, while it has been previously shown that SPCCT can be used for absolute quantification of unmixed multiple contrast agents *in vivo* [7, 8, 21–23, 32, 33], few studies have investigated the accuracy of the quantification [21, 33].

Recent *in vitro* studies have reported the quantification of iodine and gadolinium contrast agents using DECT [34, 35]. However, this technology can decompose the attenuations into at most two components because it can perform only two measurements per pixel. SPCCT, on the other hand, provides five photon-count measurements (five bins) per pixel, which theoretically allows the concomitant decomposition of the attenuations on a two- to four-material basis; two non-K-edge materials (e.g. water and iodine), and one or two K-edge materials (e.g. gadolinium and gold), given that their K-edge energies are in two different energy bins. Our results confirm this theory. In fact, we were able to show an accurate affine response of the material decomposition process both for unmixed (iodine, gadolinium and gold) and mixed (iodine–gadolinium and gadolinium–gold mixtures) solutions, despite slightly shifted offsets. Nevertheless, when two contrast agents were mixed, we observed an underestimation of the quantification of the contrast agents that was more marked for K-edge materials (e.g. gold and gadolinium). Similarly, mixing the two K-edge materials led to some cross-contamination in the iodine image. Another consequence of mixing the materials was the increase of noise and the decrease of the CNR of the materials, much more adversely affected for the K-edge materials, i.e. gadolinium and gold. Indeed, as a result we observed a twofold decrease of the CNR for the mixture of gadolinium and gold demonstrating that the four-material basis approach comes with a lower sensitivity performance limiting drastically the trade-off between contrast and noise. These outcomes can be explained by the fact that including more components in the material decomposition process means higher degrees of freedom for the maximum likelihood algorithm, leading to smaller signal per component and therefore more noise and more noise-induced bias. In addition, we observed that among the K-edge materials, the gadolinium image was more contrasted than the gold one. This can be explained by the fact that gadolinium beneficiates from a better balance of photons below and above its K-edge energy than gold in a 120-kVp beam, leading to a more accurate material decomposition.

The origins of all these imperfections can be explained by the noise on the photon counts, which propagates to noise in the decomposed sinograms and ultimately to the material maps. In addition to the inherent quantum noise, physical effects such as pulse pile-up and charge sharing as well as inaccuracies in the spectral model of the system (e.g. x-ray tube spectrum, detector response function) will further degrade the quantification results if not properly addressed in the forward model which is solved by the maximum likelihood decomposition [1, 16]. The decomposition and reconstruction algorithms are still under development. Quantification improvements are expected as more accurate models and further noise handling algorithms will be applied. For example, researchers are actively looking into including some of the still un-modelled physical effects and enhancing the accuracy of the incident spectrum and detector response function [36, 37]. Additionally, mitigating the noise would probably require using regularised iterative methods, either during the material decomposition process [38], during the reconstruction process [39] or in a one-step algorithm that would embed both processes [40].

Based on our results, and the fact that all SPCCT reconstructed images have a 100% spatial registration [13], using multiple contrast agents with different pharmacokinetics in the same biological system simultaneously can reasonably be considered. Several applications of such methods in the vascular system can be considered. For example, different contrast agents injected sequentially within a single scan could allow the imaging of multiple uptake phases of a given tissue/organ [7, 32]. It could also be possible to simultaneously visualise the vascular lumen and wall in pathologies such as atherosclerosis by using a combination of specific and non-specific contrast agents [22]. These applications would considerably enhance the diagnostic capabilities and decrease the patients’ exposure to radiation. Finally, for the past 20 years, increasing efforts have been directed toward *in vivo* imaging of gene expression and enzyme activity, particularly in oncology research. It would be interesting to develop new multi-atom contrast agents that could be modified or cleaved in targeted tissues to allow cellular activity imaging [41–43].

Today, however, the simultaneous use of multiple intravascular heavy metal-based contrast agents is still not clinically approved. Moreover, none of the K-edge candidates are approved for human use, i.e. the heavy atoms, even in the case of contrast agent-based nanoparticles. As a consequence, further pharmacological evaluations of currently available agents (e.g. concomitant use of iodine and gadolinium) or the development of new contrast media are needed.

As a study limitation, we should note that the work we report here has been done using relatively simple phantoms and a preclinical SPCCT scanner. In order to extend our study to clinical applications, we are now using a machine scaled for clinical use and assessing its spectral capabilities under varying conditions (tube currents and voltage) with phantom conditions closer to human characteristics (size, attenuation, FOV).

In conclusion, the SPCCT prototype used in this study can qualitatively and quantitatively differentiate multiple contrast agents within the same solution, opening perspectives in clinical applications. However, to fully take advantage of the SPCCT multicolour imaging performance, new agents containing K-edge materials must be developed.

## Abbreviations

AuNP: Gold nanoparticles
CI: Confidence interval
CNR: Contrast-to-noise ratio
DECT: Dual-energy computed tomography
FOV: Field of view
HU: Hounsfield units
ICC: Intraclass correlation coefficient
PBS: Phosphate buffered saline
RMSE: Root mean square error
ROI: Region of interest
SPCCT: Spectral photon-counting computed tomography
